# A Simple and Cost-Effective Method for Producing Stable Surfactant-Coated EGaIn Liquid Metal Nanodroplets

**DOI:** 10.3390/ma13173753

**Published:** 2020-08-25

**Authors:** Bingbing Xu, Feng Ye, Guangtao Chang, Ruoxin Li

**Affiliations:** College of Textile and Clothing Engineering, Soochow University, 199 Renai Road, Suzhou 215000, China; 20194015009@stu.suda.edu.cn (B.X.); 20194215002@stu.suda.edu.cn (F.Y.)

**Keywords:** liquid metal, EGaIn, nanodroplets, shaking, stable

## Abstract

Liquid metals show unparalleled advantages in printable circuits, flexible wear, drug carriers, and electromagnetic shielding. However, the efficient and large-scale preparation of liquid metal nanodroplets (LM NDs) remains a significant challenge. Here, we propose a simple and efficient method for the large-scale preparation of stable eutectic gallium indium nanodroplets (EGaIn NDs). We compared different preparation methods and found that droplets with smaller particle sizes could quickly be produced using a shaking technique. The size of EGaIn NDs produced using this technique can reach 200 nm in 30 min and 100 nm in 240 min. Benefiting from the simple method, various surfactants can directly modify the surface of the EGaIn NDs to stabilize the prepared droplets. In addition, we discovered that shaking in an ice bath produced spherical nanodroplets, and after shaking for 30 min in a non-ice bath, rod-shaped gallium oxide hydroxide (GaOOH) appeared. Furthermore, the EGaIn NDs we produced have excellent stability—after storage at room temperature for 30 days, the particle size and morphology change little. The excellent stability of the produced EGaIn NDs provides a wider application of liquid metals in the fields of drug delivery, electromagnetic shielding, conductive inks, printed circuits, etc.

## 1. Introduction

Liquid metals, as the name implies, are metals that are liquid below 300 °C [1]. Liquid metals have attracted much attention due to their good fluidity, low viscosity, and excellent electrical and thermal conductivity [2,3]. In addition, gallium-based liquid metals have unparalleled advantages in bioengineering, drug delivery, and tumor treatment because of their low toxicity and surface modification [4,5,6,7,8]. For example, Gu et al. utilized eutectic gallium indium (EGaIn) and thiolated polymers to form core–shell nanospheres, which were loaded with doxorubicin or hyaluronic acid for drug delivery and tumor treatment [9]. Miyako et al. showed that photopolymerized liquid metal nanocapsules will generate heat and active oxygen under near-infrared irradiation, and cause the transformation of liquid metal (LM), leading to the destruction of the nanocapsule, thereby controlling release of the loaded drugs [10]. Liu et al. proposed liquid metal angiography for the first time. Gallium-based liquid metal as a contrast agent was infused into the blood vessels of the heart and kidney of pigs [11]. Moreover, liquid metals have the fluidity of liquid but the conductivity of metal that is comparable with other materials including graphene, carbon nanotubes, and silver nanowires [12,13,14,15]. The films, circuits, and sensors made of liquid metals have good extensibility and flexibility, which gives liquid metals a wide range of applications in printable circuits, flexible wear, and smart sensors [16,17,18,19]. For example, Zhou et al. filled liquid metal into an elastomer sponge to prepare a highly conductive and flexible liquid metal sponge [20]. Zhang et al. used liquid metal as a filler in polymer to prepare an ultra-stretchable hydrogel [21].

However, the efficient and large-scale preparation of LM nanodroplets (NDs) remains a huge challenge [22,23,24]. Traditional methods for preparing LM NDs include sonication, high-speed shearing, microfluidic, and grinding [25,26,27,28,29,30]. The sonication method is the most widespread method for preparing LM NDs. However, the production of LM NDs by sonication has high noise and low efficiency, and it can only be prepared on a small scale in the laboratory. The high-speed shearing method can only obtain micron-level LM NDs, perhaps due to the low viscosity of the slurry and the high density of the liquid metal, while microfluidic technology requires expensive equipment and complicated operations. Although the grinding can prepare LM NDs on a large scale, it has high energy consumption, high noise, and is easily volatile during preparation.

Here, we propose a simple and efficient method for the large-scale preparation of stable EGaIn NDs. EGaIn, surfactant, defoamer, and zirconium beads were added into a sealed glass bottle. The bottle was placed in a double-layer mold, which can be filled with ice water in the outer layer, then the mold was put into a shaker. Adjusting the shaking time helps obtain nanodroplets with different particle sizes. We compared different preparation methods and found that droplets with smaller particle sizes could quickly produce by shaking. Using this technique, the size of the produced EGaIn NDs can reach 200 nm in 30 min and 100 nm in 240 min. Benefiting from the simple method, various surfactants can directly modify the surface of the EGaIn NDs to stabilize the prepared droplets. Moreover, produced EGaIn NDs have excellent stability; after storage at room temperature for 30 days, the particle size and morphology change little. It is envisaged that the method can be used as a simple and cost-effective technique to support the rapid and large-scale preparation of EGaIn NDs. Excellent stability and narrow particle size distribution give liquid metal nanodroplets unique performance in electromagnetic shielding, printable circuits, strain sensor, and bioengineering [31,32,33,34,35,36,37].

## 2. Materials and Methods 

Materials: EGaIn (Ga 75%, In 25%, melting point 16 °C) was purchased from Shenyang Jiabei Trading Co., Ltd. (Shenyang, China). The preparation of synthetic dispersant (SP) has been reported in our previous work [38]. Dispersant 190 was purchased from BYK company (Wesel, Germany). Surfactants potassium salt of isotridecanol polyoxyethylene ether phosphate (1310PK), sodium tridecyl alcohol polyoxyethylene ether sulfonate (1310SA), and sodium tridecyl alcohol polyoxyethylene ether carboxylate (1307Na) were purchased from Jiangsu Haian Chemical Co., Ltd (Nantong, China). The antifoaming agent was purchased from Evonik. The water utilized was laboratory-made deionized water.

Preparation of EGaIn NDs: First, 1 g of EGaIn, 1 g of SP, and a small amount of defoamer were added into a bottle that was filled with deionized water to a total weight of 50 g and 300 g of zirconium beads (0.3–0.4 mm). The bottle was placed in a double-layer mold which can be filled with ice water in the outer layer. Then, the mold was put into a shaker (SK550, FFM, Sassenheim, Netherlands), and the shaking time was adjusted from 10 to 240 min. When the shaking process was finished, we used a paper funnel (mesh size: 150) to separate zirconium beads from NDs. In this work, different preparation methods are studied, as well as different dispersants, the ratio of polymer to liquid metal, storage time, and so on.

Characterization: A particle size analyzer (Nanotrac wave II, Microtrac, Krefeld, Germany) was used to measure the size and distribution of the LM NDs. Scanning electron microscopy (SEM, S-4800, Hitachi, Tokyo, Japan) was used to observe the particle size and morphology of the nanoparticles. Transmission electron microscopy (TEM, Tecniai G2 F20 S-TWIN, Hillsboro, OR, USA) and energy-dispersive X-ray spectroscopy (EDS, Tecniai G2 F20 S-TWIN, Hillsboro, OR, USA) were used to observe the size, morphology, and element distribution of the nanodroplets. 

## 3. Results and Discussion

**Figure 1:**
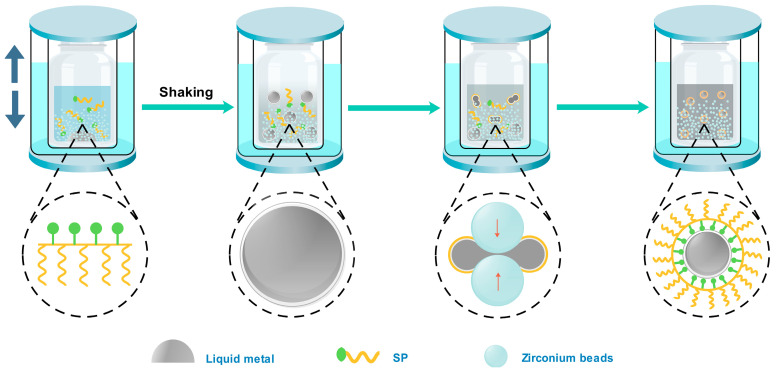
Schematic of the preparation of liquid metal nanodroplets (LM NDs) by shaking.

The production process of stable EGaIn NDs is schematically illustrated in Figure 1. First, 1 g of EGaIn, 1 g of SP, and a small amount of defoamer were added into a bottle that was filled with deionized water to a total weight of 50 g and 300 g of zirconium beads (0.3–0.4 mm). The bottle was placed in a double-layer mold that can be filled with ice water in the outer layer. Then, the mold was put into a shaker. During the production process, the bulk liquid metal was broken into microdroplets due to the collision between zirconium beads. With additional shaking, the microdroplets are continuously collided by the zirconium beads, and all of them gradually turn into NDs. The outer layer of the NDs is covered into gallium oxide, mainly because gallium is easier to oxidize in water than indium. The rapid formation of a thin oxide layer on the surface of EGaIn NDs helps to prevent coalescence back into bulk EGaIn and also prevents further oxidation of the gallium inside [25]. However, there is not enough stability for the oxide layer to stabilize the EGaIn droplets within aqueous media. Therefore, the appropriate dispersant is necessary in the production process of EGaIn NDs. In this work, we used a series number of surfactants to stabilize the NDs and found the branched polymer with carboxyl group in the main chain performed better. The gallium oxide formed around the nanodroplets provides anchor points for the carboxyl groups in the dispersant [39]. The carboxyl group and gallium oxide are tightly bound together, so that the dispersant forms a dense protective layer around the droplets, which prevents adjacent droplets from gathering and keeps the droplets stable during storage [17,40,41,42].

In contrast to other preparation methods, shaking has the advantages of being fast, efficient, and low-cost [25,42]. As shown in Figures S1 and S2, it is clear that the high-speed shearing and sonication bath have a poor dispersion of liquid metals, since microdroplets still exist after 240 min. Sonication probe and grinding only produce droplets of more than 200 nm after processing for 240 min. Microfluidic technology obtained droplets of more than 100 nm in a short time, but the equipment is expensive and the operation is complicated, which can only be produced in small batches. Shaking demonstrates unparalleled advantages, not only producing nanodroplets quickly and efficiently, but also with low cost that can be prepared on a large scale. 

**Figure 2:**
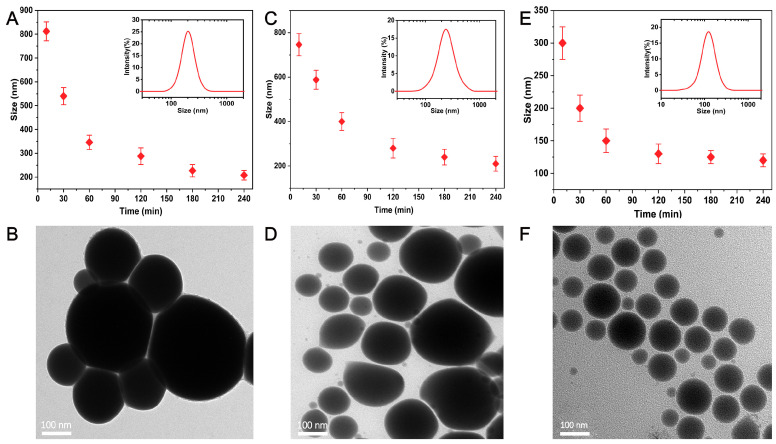
Particle size of eutectic gallium indium nanodroplets (EGaIn NDs) with different (**A**) Sonication probe, (**C**) Grinding, and (**E**) Shaking times. The insets show the corresponding size distribution of EGaIn NDs after processing for 240 min. TEM images of EGaIn NPs after 240 min of (**B**) Sonication probe, (**D**) Grinding, and (**F**) Shaking.

**Figure 3:**
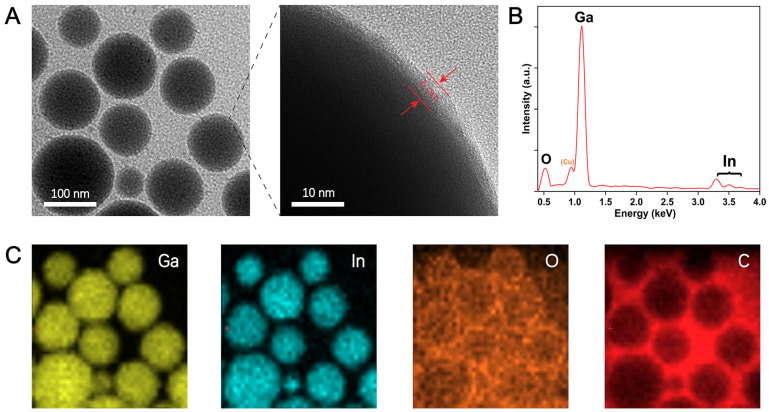
(**A**) TEM image and (**B**) energy-dispersive X-ray spectroscopy (EDS) spectrum of EGaIn NDs obtained after shaking for 240 min. (**C**) EDS mapping of gallium, indium, oxygen, and carbon for the EGaIn NDs.

In order to better illustrate the effect of processing time on particle size, we conducted a series of experiments to compare the preparation of EGaIn NDs using shaking with other methods. Figure 2a shows the relationship between particle size and process time, the inset shows the size distribution of EGaIn NDs after processing for 240 min by sonication probe. It is clear that the particle size is more than 800 nm after 10 min of sonication probe and more than 200 nm after 240 min (Table S1). Figure 2b shows TEM images of EGaIn NDs after 240 min of sonication probe. Figure 2c,d show that the particle size reached about 700 nm after 10 min of grinding, and it reduced to about 200 nm after 240 min. Surprisingly, nanodroplets of about 300 nm were obtained after 10 min of shaking, and nanodroplets of about 100 nm were obtained after 240 min (Figure 2e,f). It is obvious that the droplet size does not decrease significantly as the shaking time increases after 120 min. This is because as the size of the NDs decreases, more and more energy is required to break them. Moreover, when the size of the NDs particle size decreases to a certain degree, the energy input is used to break the aggregation between the NDs and cannot continue to reduce the size of the NDs, which is a dynamic equilibrium process of the whole system, and the size of the NDs also tends to saturate. Figure 3a shows the TEM image of EGaIn NDs prepared by shaking for 240 min; the particle size of EGaIn NDs is about 100 nm, and there is a bright thin layer on the surface of the droplet. From the enlarged image, it can be seen that the thickness of the bright thin layer is about 5 nm, which is similar to the reports in the literature [25]. We obtained an EDS spectrum and mapping to further analyze nanodroplets’ composition and element distribution. As shown in Figure 3b,c, gallium and indium are evenly distributed in the droplets, and oxygen and carbon accumulate on the surface of the droplets, which is mainly because of the oxide layer on the surface of the liquid metal and the coated polymer. There is also a lot of carbon in the blank background because the copper mesh we used is coated with a thicker carbon film. From these results, it can be seen that the EGaIn NDs prepared by shaking have a small particle size and a regular morphology. The outer layer of the coated polymer not only prevents the adjacent droplets from becoming larger, but also makes the droplets stable during storage. Consequently, shaking is a fast, efficient, and cost-effective method for preparing EGaIn NDs.

Benefiting from the simple method, we further investigated different dispersants, including 1310SA, 1307Na, 1310pk (the corresponding chemical structural formula are shown in Figure S3), BYK190, and SP. As shown in Figures S4 and S5, EGaIn NDs prepared with 1310SA or 1307Na had a large particle size and were extremely unstable. Furthermore, precipitation accumulated after a period of time. The size of EGaIn NDs prepared with 1310 PK or BYK 190 can reach 100–200 nm, but the stability was not particularly good, and obvious precipitation occurred after 10 days. Interestingly, we found that without adding surfactant, only adjusting the pH of the solution achieved the dispersion effect as well [43,44]. As shown in Figure S5, we adjusted the pH of the solution, the size of the EGaIn NDs prepared by shaking of 120 min is less than 400 nm, but after a short period of time, delamination and precipitation occurred. The experimental results indicate that dispersants play an important role in the production and storage of EGaIn NDs. All factors considered, we selected the SP dispersant to produce EGaIn NDs. Furthermore, we explored the effect of SP content on droplets preparation. First, 1 g of EGaIn and a small amount of defoamer were added into a bottle that was filled with deionized water to a total weight of 50 g; then, different amounts of SP are added (0.1, 0.2, 0.5, 1, and 2 g). Finally, we added 300 g of zirconium beads (0.3–0.4 mm) and shook for 120 min. As shown in Figure S6, the particle size of the produced droplets decreases as the amount of SP increases. With less SP content, there is not enough dispersant to stabilize the droplets, and the droplet size is larger. A large number of bubbles are generated when too much SP is added, which affects the efficiency of shaking. Therefore, the ratio of SP to liquid metal of 1:1 was adopted in this work.

**Figure 4:**
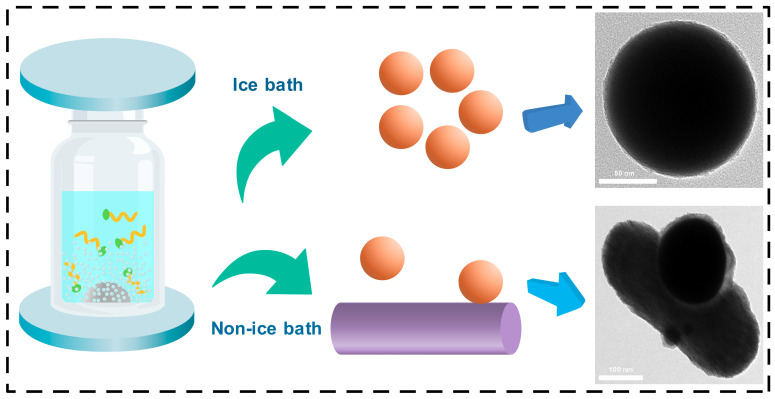
The schematic and corresponding TEM images of shaking in an ice bath and non-ice bath.

We noticed that temperature has a significant influence on the preparation of EGaIn NDs. At the beginning, we started the shaking process for 120 min without cooling water. The color of the EGaIn NDs produced through this was not conventional gray, but rather an unusual black. In order to confirm the size and morphology of the droplets, we further obtained SEM images. As shown in Figure S7, spherical EGaIn NDs are not visible; they are replaced by rod-like and irregularly shaped particles. We supposed that because the temperature is too high, gallium oxide gradually converted to GaOOH [42]. Therefore, we repeated the same experiment in the ice bath. Figure 4 depicts the schematic and corresponding TEM images of shaking in the ice bath and non-ice bath. It can be seen from the TEM images that under the conditions of the ice bath, the EGaIn NDs produced are spherical particles with a diameter of about 100 nm. However, under non-ice bath conditions, spherical particles gradually transformed into rods after 30 min of shaking. Moreover, as the shaking time increased, more rod-shaped particles appeared, which is attributed to the fact that gallium is more reactive than indium and performs to form gallium oxide in water [27,45,46]. During the shaking, the zirconium beads collide with each other, generating a large amount of heat, and the gallium oxide on the surface of the droplets will gradually transform into gallium oxide hydroxide [47]. Consequently, there are relatively few gallium oxides in the TEM image where the spherical particles and rod-shaped particles are in contact. This morphology and limited oxidation phenomenon also verify our previous conjecture.

**Figure 5:**
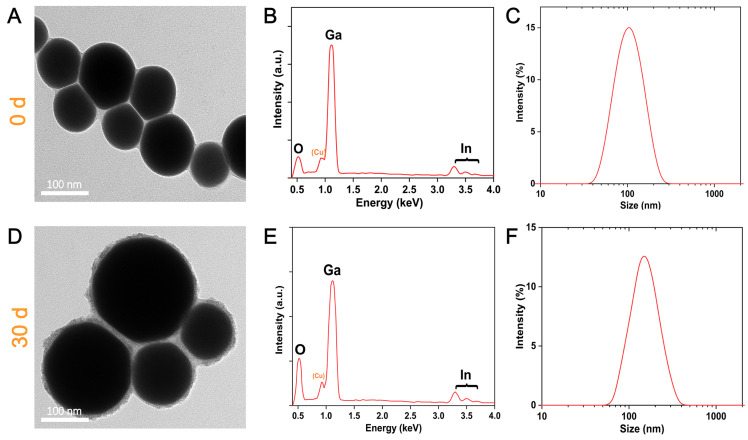
(**A**) TEM image, (**B**) EDS spectrum, and (**C**) Size distribution of EGaIn NDs after 0 days. (**D**) TEM image, (**E**) EDS spectrum, and (**F**) Size distribution of EGaIn NDs over a period of 30 days.

After obtaining stable and small-size EGaIn NDs, we further explored the stability of the droplets in water for a longer period of time. Figure 5a shows the morphology and particle size of the freshly prepared droplets. It is clear that the droplets are regular spherical particles with a thin oxide layer and polymer coating around it. The EDS spectrum (Figure 5b) shows less oxygen content, which indicates that the surface layer of the droplet contains less gallium oxide, and Figure 5c shows the droplets size distribution and average particle size (100 nm). After storing at room temperature for 30 days, we further obtained the TEM image of the droplets, as shown in Figure 5d. The particle size of the droplet increased a little, and a thick and rough shell appeared around the droplet. This morphology is due to the fact that during the long-term storage process, although the polymer forms a dense layer around it to protect the internal gallium from further oxidation, this only slows down the oxidation process rather than completely eliminating oxidation. Inevitably, gallium will come into contact with water or air to form gallium oxide, as evidenced by the EDS spectrum (Figure 5e). The particle size distribution after 30 days is depicted in Figure 5f, in which the peak of the distribution is shifted by 30 nm toward the large size; this result is also consistent with our previous research [38]. We believe that if protective gas such as nitrogen is passed into the produced EGaIn NDs, the storage time will be longer.

Such a simple, efficient, and cost-effective method produces stable EGaIn NDs with a narrow particle size distribution and lays a solid preparation foundation for the application of LM NDs, making it widely used in bioengineering, drug delivery, conductive ink, printed circuit, and other fields [48,49,50,51,52]. Depending on the applications, we can obtain droplets with different particle sizes by adjusting the shaking time. We can even quickly obtain GaOOH by this method [46]. However, there are still shortcomings in this process that must be addressed. We simply explained that pH and temperature have a great influence on the preparation and particle size of the droplets, without detailed discussion. Future work could optimize the pH and temperature when preparing LM NDs, prepare stable LM NDs in different solvents, and obtain LM NDs with a uniform particle size by gradient centrifugation.

## 4. Conclusions

In summary, this article reports a simple, efficient, and cost-effective method for preparing stable liquid metal nanodroplets. We compared different preparation methods and found that droplets with smaller particle sizes could quickly produce by shaking EGaIn NDs of about 300 nm in 10 min and about 100 nm in 240 min. Benefiting from the simple method, various surfactants can directly modify the surface of the EGaIn NDs to stabilize the prepared droplets. In addition, we discovered that shaking in an ice bath produced spherical nanoparticles, and after shaking for 30 min in a non-ice bath, rod-shaped GaOOH appeared. Furthermore, produced EGaIn NDs have excellent stability, after storage at room temperature for 30 days, the particle size and morphology change little. This simple, efficient, and cost-effective method provides a wider application of liquid metals in the fields of drug delivery, conductive inks, printed circuits, etc.

## Supplementary Materials

The following are available online at https://www.mdpi.com/1996-1944/13/17/3753/s1, Figure S1. Particle size of EGaIn NDs prepared by different methods; Figure S2. Images of suspension for EGaIn NDs produced by different methods; Figure S3. Chemical structural formulas of 1310PK, 1310SA, 1307Na and SP; Figure S4. Particle size of EGaIn NDs produced with different dispersants; Figure S5. Images of suspension for EGaIn NDs produced with different dispersants. (pH = 3 means no additives, only adjusting the pH = 3 of the solution); Figure S6. Effect of SP content on the particle size of EGaIn NDs; Figure S7. SEM image of EGaIn NDs after shaking in non-ice bath for 240 minutes; Table S1. Particle size and PDI of EGaIn NDs prepared by shaking, grinding, and sonication probe for 240 min.
