# Supplementary material for: A Simple and Cost-Effective Method for Producing Stable Surfactant-Coated EGaIn Liquid Metal Nanodroplets
Source: Materials (Basel). 2020 Aug 25;13(17):3753. doi: 10.3390/ma13173753 (PMC7503906; doi:10.3390/ma13173753)
Supplement: Supplementary file 1 [file materials-13-03753-s001.pdf]

materials

Supplementary Information

A Simple and Cost-Effective Method for Producing Stable Surfactant-Coated EGaIn Liquid Metal Nanodroplets

Bingbing Xu, Feng, Ye, Guangtao Chang*, and Ruoxin Li*

We chose SP as a dispersant and used different methods to prepare EGaIn NDs, including shearing (SFJ-500, 5000rpm, Shanghai, China), sonication bath (SB-5200DTD, 300w, Ningbo, China), sonication probe (FS-1200N, 800w, Shanghai, China), grinding (SFJ-500, 5000rpm, Shanghai, China), microfluidic (NanoGenizer, 30000 psi, Los Angeles, USA), shaking (SK550, Sassenheim, Netherlands). The ratio of SP to liquid metal of 1:1 was adopted in this work.

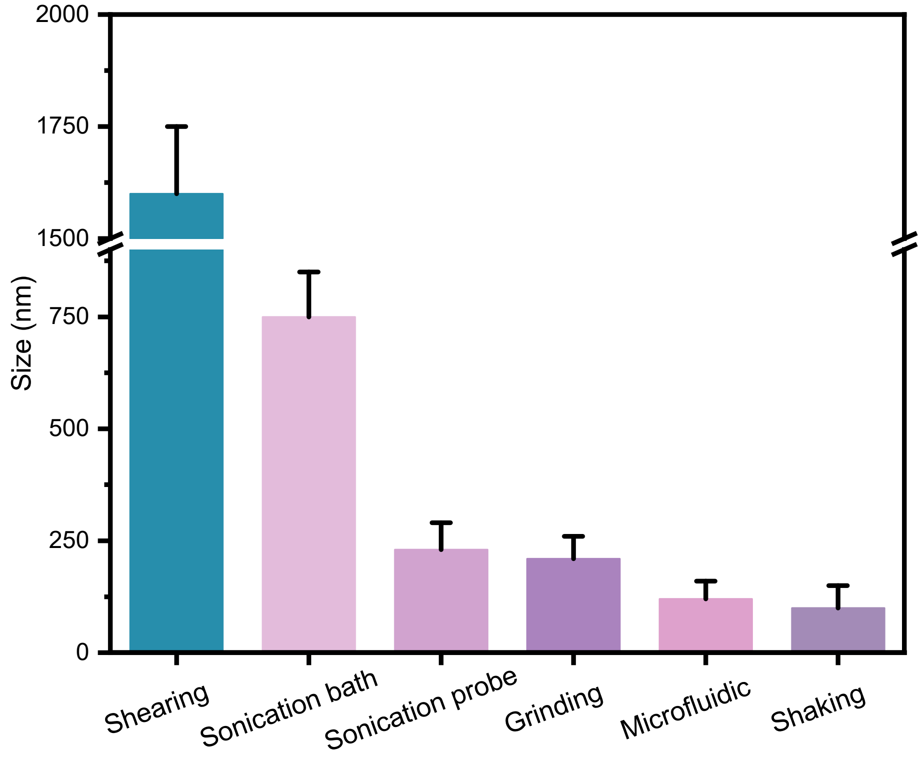

Figure S1. Particle size of EGaIn NDs prepared by different methods.

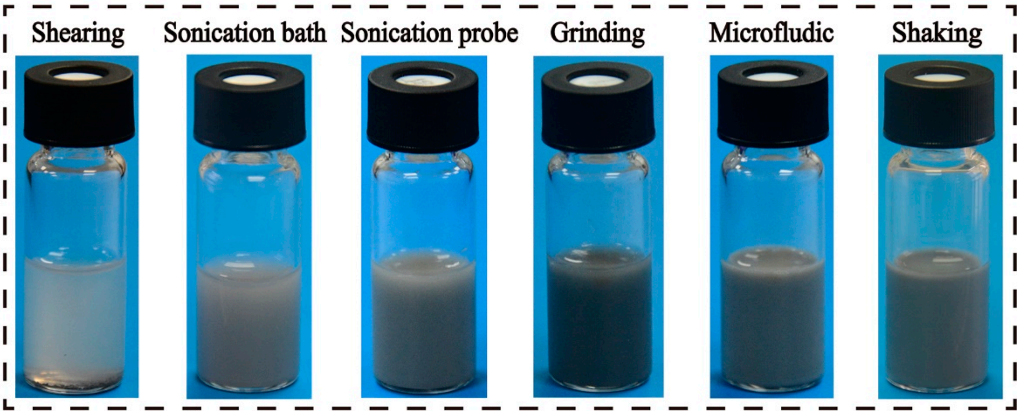

Figure S2. Images of suspension for EGaIn NDs produced by different methods.

Table S1. Particle size and PDI of EGaIn NDs prepared by shaking, grinding, and sonication probe for 240 min.

	Shaking			Grinding			Sonication probe		
	1	2	3	1	2	3	1	2	3
Size (nm)	104	107	101	226	208	219	258	243	252
PDI	0.12	0.14	0.11	0.21	0.22	0.19	0.23	0.24	0.26

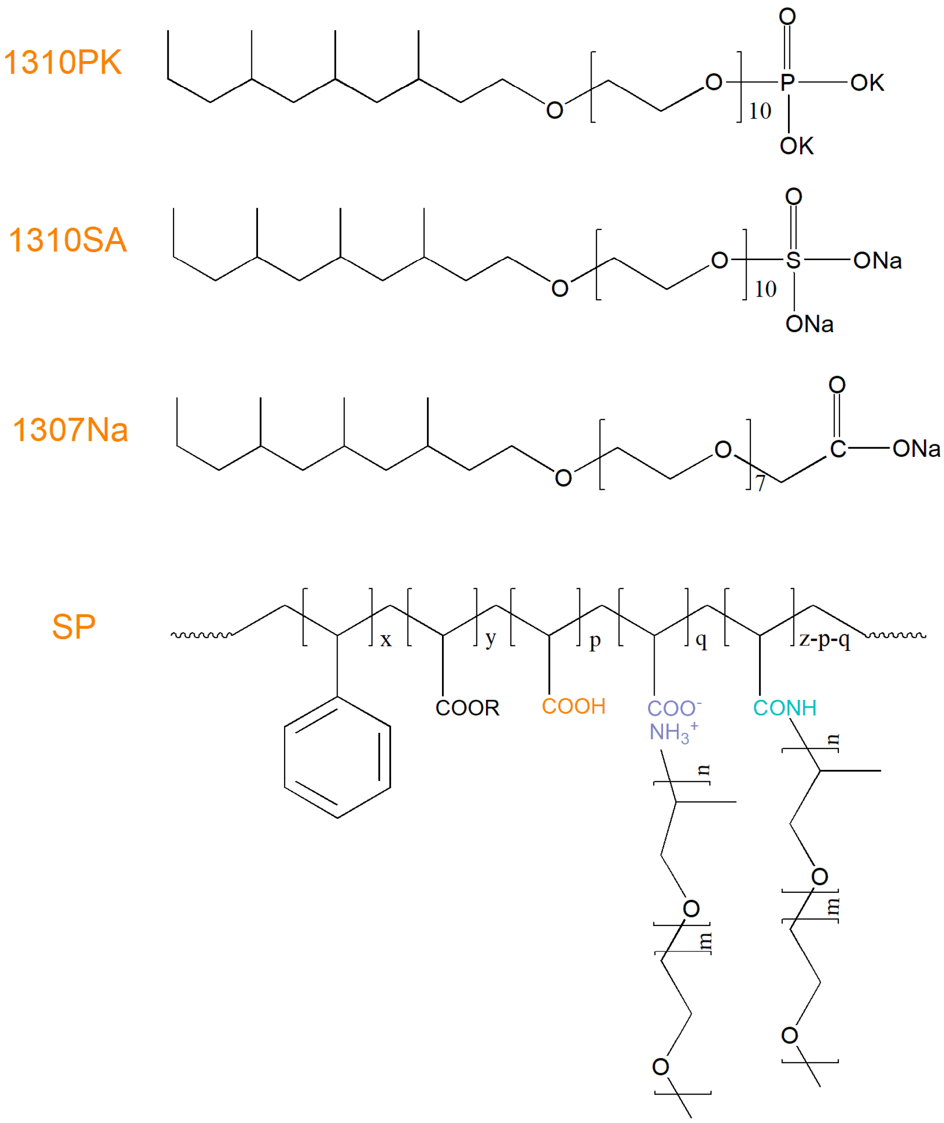

Figure S3. Chemical structural formulas of 1310PK, 1310SA, 1307Na and SP.

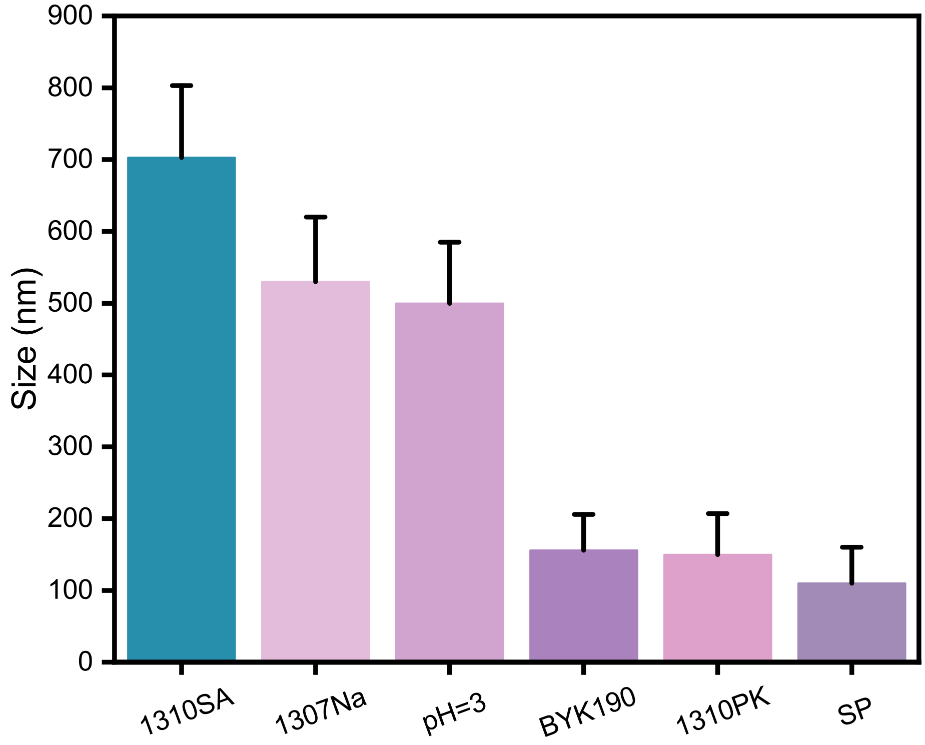

Figure S4. Particle size of EGaIn NDs produced with different dispersants.

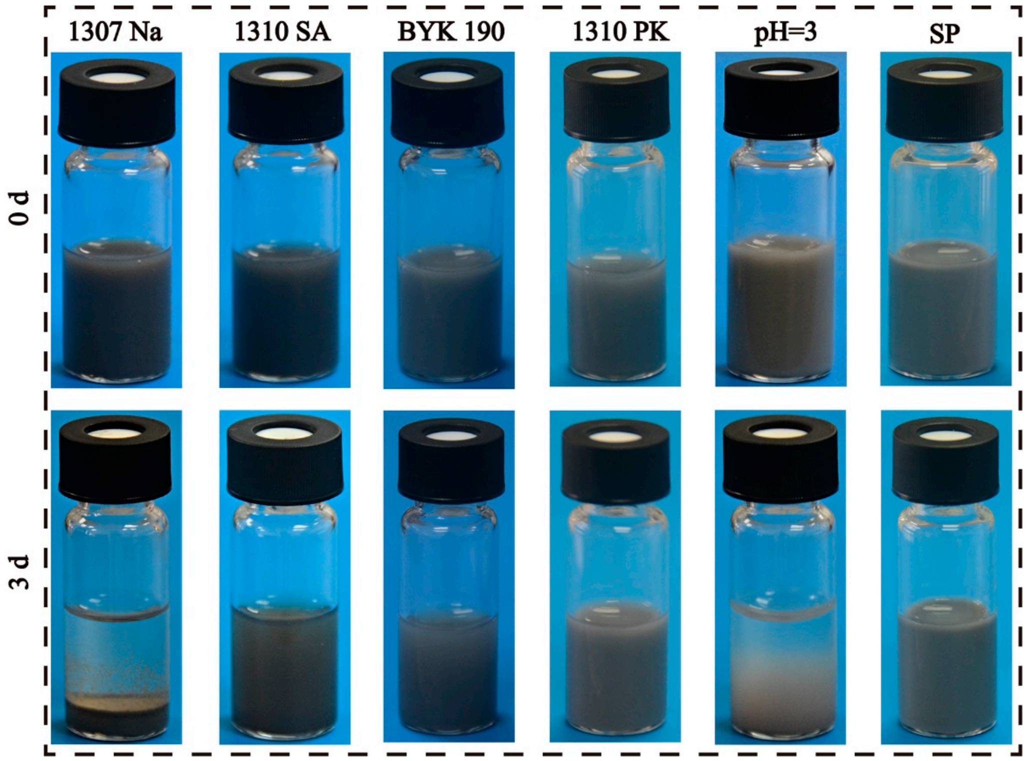

Figure S5. Images of suspension for EGaIn NDs produced with different dispersants. (pH=3 means no additives, only adjusting the pH=3 of the solution)

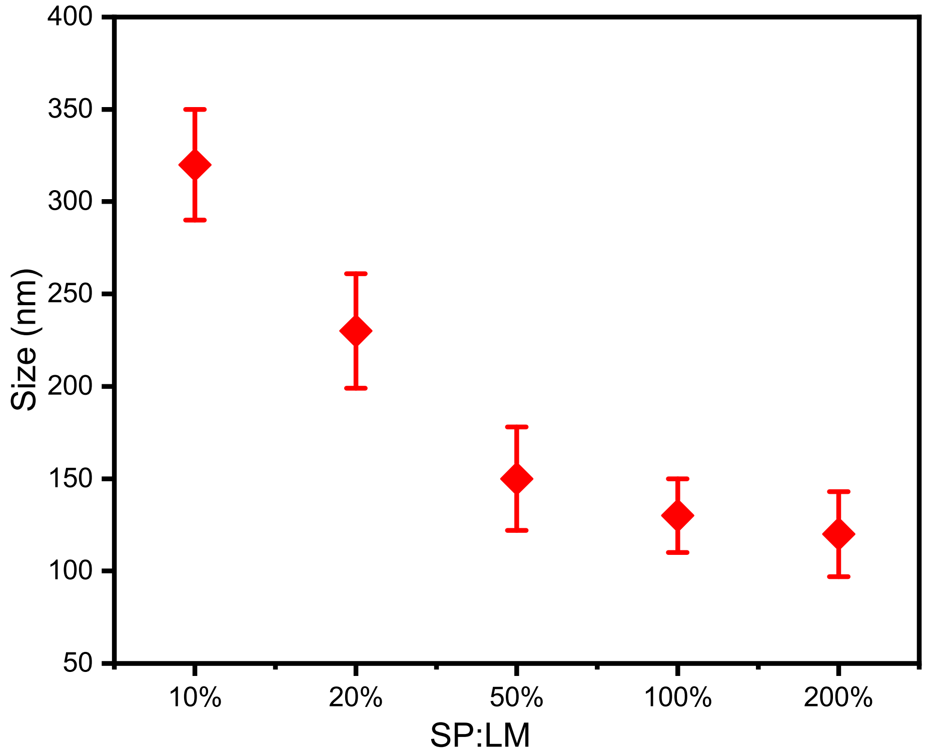

Figure S6. Effect of SP content on the particle size of EGaIn NDs

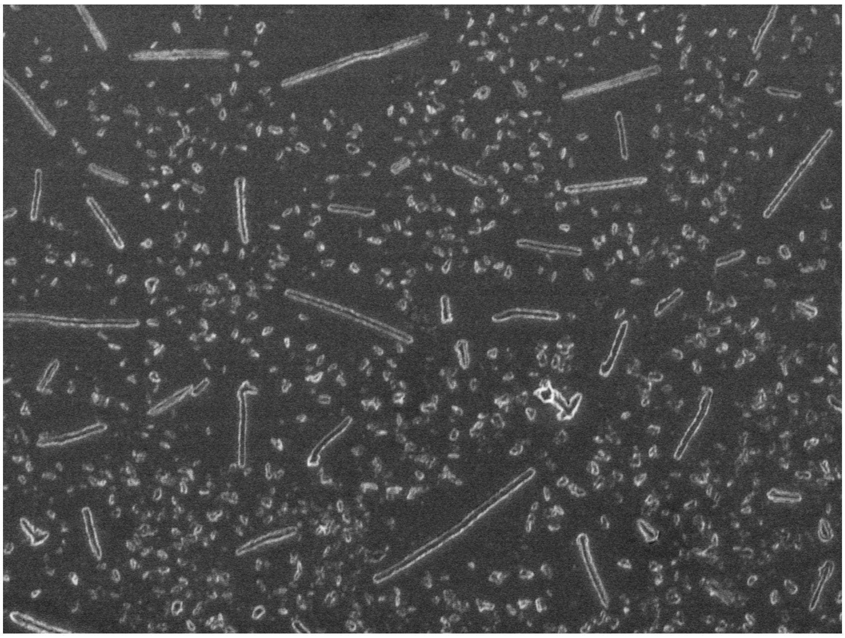

Figure S7. SEM image of EGaIn NDs after shaking in non-ice bath for 240 minutes
